# Cluster analysis of behavioural and event-related potentials during a contingent negative variation paradigm in remitting-relapsing and benign forms of multiple sclerosis

**DOI:** 10.1186/1471-2377-11-64

**Published:** 2011-06-02

**Authors:** Javier J Gonzalez-Rosa, Manuel Vazquez-Marrufo, Encarnacion Vaquero, Pablo Duque, Monica Borges, Carlos M Gomez-Gonzalez, Guillermo Izquierdo

**Affiliations:** 1 Department of Experimental Psychology, University of Seville, Seville, Spain; 2 Multiple Sclerosis Unit, Virgen Macarena Hospital, Seville, Spain

## Abstract

**Background:**

Event-related potentials (ERPs) may be used as a highly sensitive way of detecting subtle degrees of cognitive dysfunction. On the other hand, impairment of cognitive skills is increasingly recognised as a hallmark of patients suffering from multiple sclerosis (MS). We sought to determine the psychophysiological pattern of information processing among MS patients with the relapsing-remitting form of the disease and low physical disability considered as two subtypes: 'typical relapsing-remitting' (RRMS) and 'benign MS' (BMS). Furthermore, we subjected our data to a cluster analysis to determine whether MS patients and healthy controls could be differentiated in terms of their psychophysiological profile.

**Methods:**

We investigated MS patients with RRMS and BMS subtypes using event-related potentials (ERPs) acquired in the context of a Posner visual-spatial cueing paradigm. Specifically, our study aimed to assess ERP brain activity in response preparation (contingent negative variation -CNV) and stimuli processing in MS patients. Latency and amplitude of different ERP components (P1, eN1, N1, P2, N2, P3 and late negativity -LN) as well as behavioural responses (reaction time -RT; correct responses -CRs; and number of errors) were analyzed and then subjected to cluster analysis.

**Results:**

Both MS groups showed delayed behavioural responses and enhanced latency for long-latency ERP components (P2, N2, P3) as well as relatively preserved ERP amplitude, but BMS patients obtained more important performance deficits (lower CRs and higher RTs) and abnormalities related to the latency (N1, P3) and amplitude of ERPs (eCNV, eN1, LN). However, RRMS patients also demonstrated abnormally high amplitudes related to the preparation performance period of CNV (cCNV) and post-processing phase (LN). Cluster analyses revealed that RRMS patients appear to make up a relatively homogeneous group with moderate deficits mainly related to ERP latencies, whereas BMS patients appear to make up a rather more heterogeneous group with more severe information processing and attentional deficits.

**Conclusions:**

Our findings are suggestive of a slowing of information processing for MS patients that may be a consequence of demyelination and axonal degeneration, which also seems to occur in MS patients that show little or no progression in the physical severity of the disease over time.

## Background

In most cases, the onset of multiple sclerosis (MS) is marked by relapses and remissions, termed relapsing-remitting MS (RRMS), and the majority of patients with this MS subtype are characterized by a continuous accumulation of disability [1]. On the other hand, there is a minority of patients who are characterized by having few relapses at the onset of the disease and an accumulation of modest or no disability over a long period of time, so they are often described as having a benign course of MS (BMS). However, this benign prognosis is currently being discussed [2,3].

Cognitive dysfunction is a frequent finding in patients with MS [4-6]. Deficits in memory, attention, speed of information processing and executive functions are typical and about 30% to 70% of patients experience impairment in these cognitive domains during the course of the disease [4-6]. Longitudinal studies have consistently revealed a progression of cognitive impairment over time in numerous MS disease courses [7,8] even in the absence of clinical disability [9,10].

During the last few decades, cognitive assessment using scalp-recorded event-related brain potentials (ERPs) have proved to be an adequate method and an objective tool to observe cognitive-related aspects in different neurological diseases. ERPs permit a precise analysis of the time course of neural events supporting task performance and can reveal both psychophysiological correlates of poor performance, providing a unique insight into information processing.

Whereas early-latency components of ERPs (N1 and P2) can be used to assess the first stages of information processing [11,12], the long-latency components of ERPs (N2, P3 and late deflections after P3) can provide a more sensitive method for assessing higher-order information processing [11-13]. Therefore long-latency components have been used more frequently than early-latency components to evaluate the cognitive complaints of most neurological diseases. In cognitive-oriented ERPs studies, the so-called contingent negative variation (CNV) is of particular interest. The CNV is an event-related slow negative shift in the human scalp electroencephalogram (EEG) that occurs between two successive stimuli (a cue stimulus and an imperative stimulus) that are associated with or are 'contingent' on each other. It is thought that the CNV is able to mirror certain aspects of motor and cognitive preparation and endogenous attention [14-16].

It has been previously shown that MS patients display enhanced latency in both early and late ERP components, whereas the amplitude of ERP may also be considerably reduced in the final stages of the disease. The main studies with MS patients have reported abnormalities in the latency or the amplitude of late ERP components, such as N2 or P3 [17-20], but also in early ERP components such as N1 or P2 [17,18,21]. These results have been interpreted, and supported by neuropsychological and neuroimaging evidence, as a general objective index of cognitive impairment in MS patients, characterized by slow information processing, as a result of disconnections between cortical-subcortical and cortico-cortical structures [17,18,22-24].

Most of these results using ERPs in MS disease have been elicited during discriminatory tasks, classically oddball tasks. However, new studies using more refined tasks may explore other impaired cognitive domains or detailed aspects in the information processing from a psychophysiological approach [24]. In particular, the study of attention mechanisms using the central cue Posner paradigm [25,26] could be a powerful tool to explore basic processes underlying more complex information processing skills in these patients. The central cue Posner paradigm allows studying the deployment of attention to certain positions in space [16,27] from a behavioural and psychophysiological point of view.

The motivation for the present study was to investigate the information processing patterns and abnormalities of attentional mechanisms associated with the Posner paradigm both in benign and typical relapsing-remitting forms of MS disease. To our knowledge, there are no published studies regarding the temporal dynamics of CNV associated to a Posner task in MS disease. According to previous psychophysiological studies [28,29], we suggest that patients with a benign profile of MS also manifest altered cognitive processing that may be different from that which patients with a typical relapsing-remitting course demonstrate. Furthermore, and consistent with current evidence about the natural course of MS disease [1,2], we suggest that the deterioration of different cognitive mechanisms is masked throughout the relapsing-remitting course of MS despite minimal physical disability. Additionally, and in an effort to explore natural subgroups of MS patients from a different perspective, we conducted a cluster analysis to establish particular grouping of individuals on the basis of their common psychophysiological pattern and cognitive profile and whether this particular grouping might also be related to clinical parameters.

## Methods

A previous paper [28] incorporates subsets of the data presented here, focusing on the contribution to preliminary psychophysiological results in different groups of MS disease. The current study incorporates new in-depth psychophysiological measures as well as more refined statistical analysis in order to identify a relationship between the studied measures and the identification of natural subgroups based on psychophysiological measures.

### Participants

Clinical, behavioural and ERP data were collected from twenty-seven MS patients, clinically defined according to the Poser criteria [30], and classified in two clinical subgroups: (1) seventeen had typical relapsing-remitting MS (RRMS), defined as a history of several relapses and remissions and with disability on the Kurtzke Expanded Disability Status Scale (EDSS) [31] less than 3.5; (2) ten had a profile of benign MS (BMS), defined by a relapsing-remitting onset, but a disease course of more than 8 years without relapses and remissions and with an EDSS of less than 3. Other inclusion criteria of our study were: clinical remission of the disease in the last three months prior to the study; and absence of corticosteroid treatment for at least three months. Patients were excluded if: a major depressive disorder had begun after the onset of MS, if patients showed any other CNS disorder, or metabolic, psychiatric, learning disorders; or in the presence of physical impairment (motor and visual) that might have interfered with testing. Lastly, we must highlight that overall, the MS patients included had no immunomodulation record before beginning our study.

Patients were previously assessed at the Multiple Sclerosis Unit of the Neurology Unit of the Virgen Macarena Hospital (Seville, Spain) and participated voluntarily in the psychophysiological testing. All participants signed informed consent before their inclusion in the study and the protocol was approved by the ethics committees of the participating hospital and the University of Seville. Participants included in this study formed the same dataset as that used in Gonzalez et al, 2006. MS patients were matched with a healthy control group for age, sex and educational level with a healthy control group. In addition to these variables, age at disease onset, disease duration, and EDSS scores were collected for all MS patients. Table 1 details the demographic and clinical MS variables of all participants in this study.

**Table 1:** Demographic and MS clinical variables of all participants

		*Groups*		
	
	CONTROLS	RRMS	BMS	*p*
*N*	18	17	10	- - -
*Sex(female)*	15 (83%)	12 (80%)	6 (60%)	0.716
*Education (years)*	10.67 ± 2.3	9.9 ± 3.9	9.2 ± 3.1	0.537
*Age*	36.54 ± 8.73 [26-54]	38.88 ± 9.04 [24-63]	42.30 ± 7.21 [28-56]	0.247
*Age at onset*	- - -	34.12 ± 6.5 [23-44]	30.90 ± 6.9 [21-42]	0.414
*EDSS*	- - -	2.1 ± 1.3 [0-3,5]	1.6 ± 0.9 [1-3]	0.609
*Disease Duration*	- - -	4.67 ± 4,13 [1-18]	12.09 ± 4.77 [8-16]	**0.001**

Mean, standard deviation, and range are shown for age, age at onset, disease duration and EDSS score. Sex (% female) and years of education are also shown.

### Stimuli and Procedures

All stimuli were presented on a black computer screen that was viewed from a 70-cm distance. Subjects sat in a chair in front of the video monitor displaying the visual stimuli in a sound-attenuated room with dimmed lights. They were asked to fix their eyes on the central fixation (a white cross) and to remain as quiet as possible. Participants were asked to detect and respond to a visual target stimulus (a black and red checkerboard) and to ignore and not respond to a standard visual stimulus (a black and white checkerboard). Both stimuli were preceded by a central directional cue.

**Figure 1:**
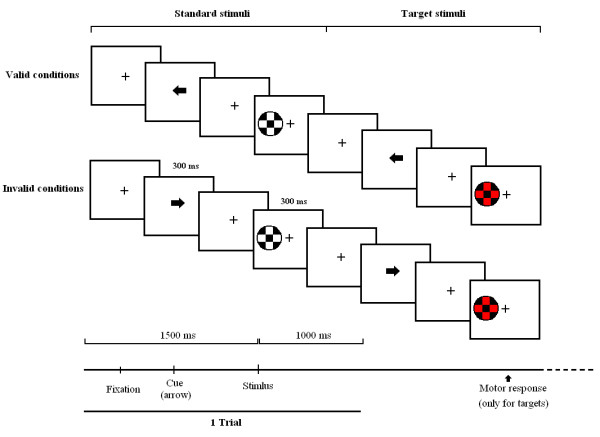
**Experimental paradigm**. A modified version of the central-cue Posner's paradigm. 75% standard stimuli, 25% target trials. Five blocks with 200 trials each were presented. All stimuli last for 300 milliseconds (ms). The presentation of stimuli was random (left or the right side). Target and Standards were presented at 2.46 degrees to the left or the right of the central fixation point. Cues could point towards the position where the stimulus appears (valid trials, 80%) or the opposite side (invalid trials, 20%). The subject's task was to indicate the appearance of the target stimulus in the left or right visual field by pressing the left button or right button. Therefore, left valid, left invalid, right valid and right invalid trials appeared both for standard and target trials.

A detailed description of the experimental paradigm can be found in references 26 and 28. For a basic description please see the footnote in Figure 1.

### ERP recording procedures and measurements

EEG recordings were obtained using 13 scalp sites from an electrode cap (Electrocap™) according to the International 10-20 electrode system. Blinks and saccades were controlled using vertical and horizontal electrooculogram (VEOG and HEOG) and were recorded with bipolar recording by means of electrodes situated in the external canthi of the ocular orbits and in the inferior and superior positions of the left orbit. All electrodes were referenced to the left mastoid and re-referenced off-line to the linked mastoid and the ground electrode was placed on the mid-forehead. The amplification gain was 20,000 (Neuroscan, Grass) and data were filtered using a band-pass of 0.01-100 Hz. EEG was digitised at a frequency rate of 500 Hz and the impedance was kept below 5 kOhm. Previous to average calculation, each EEG epoch was visually inspected and epochs containing excessive movement or EEG artefacts were rejected. Additionally, trials in which HEOG artefacts were higher than ± 60 μV were rejected. The period of 100 ms previous to standard/target stimuli was considered as the baseline for standard/target ERP analysis. A new baseline of 100 ms prior to the cue stimuli was computed for the CNV analysis. The different experimental conditions were averaged independently.

#### Behavioural measures

Various measures were recorded to assess behavioural performance for each participant. Reaction times (RTs) to each target presentation and the percentage of correct responses (% CRs) were obtained. The percentage of error (% Errors) was also analysed. An error was considered if: no response to the target occurred, or the response occurred 700 ms after target stimulus (*missed*); when responses to the targets occurred with the wrong finger (*target error*); and when the response was to standard stimuli (*false positive*). For the percentage Error analysis (due to great ratio of valid stimuli in comparison to invalid stimuli), the difference between number of stimuli of each condition were computed using a ratio-adjusted percentage.

#### ERP measures

ERP components were functional and operationally defined on the basis of previous research, in terms of experimental task effects in a specified latency range and in specific scalp locations. For all ERP components the variables considered were the baseline-to-peak amplitudes and the stimulus-to-peak latencies. These measured time windows were determined on the basis of inspection of the grand average waveforms in the control group. The amplitude of each ERP component was measured within latency windows centred on the peak latency of the grand average ERPs.

In order to determine relevant time windows of amplitudes and latencies of the ERP components, peak analysis was separately performed for the standard and target stimuli and during the CNV period. Thus, for standard stimuli, early N1 (eN1), P1, N1, P3 and late negativity (LN) components were measured. Different ERP components were only analyzed when they appeared. In the case of target stimuli, it was not possible to analyze P1 and N1, probably due to the lack of an adequate signal-to-noise ratio, given the low number of target stimuli. However, P2, N2, P3 and LN were measured. Each ERP component was obtained for each subject as the greatest positive (P1, P2, P3) or negative (N1, N2, LN) deflection on typical electrodes in the appropriate time window (for an example of procedure, see Gonzalez-Rosa et al, 2006). LN component was defined as the highest negative deflection between 400 and 500 ms immediately after the P3 wave on the ERP, and amplitude was measured at frontal, central and parietal electrodes from *difference *waveforms obtained by subtracting invalid minus valid trials of average ERPs both in the target and standard condition.

For the measurement of CNV, three areas of interest were defined at three different time windows and their amplitudes were calculated with respect to a baseline (100 ms prior to the cue stimulus). Immediately after the sensory ERPs induced by the cue stimulus, an acute negativity between 400 and 600 ms was presented. This early or initial phase of the CNV was termed eCNV and was defined as the highest negative deflection at this time window on central and parietal scalp locations. The central phase of the CNV (cCNV) was defined as the mean negative amplitude on fronto-central electrodes between 600-1000 ms after the cue stimulus. On the late part of the CNV, a slow negative potential appeared during the time preceding the imperative stimuli. This late or terminal period of the CNV (tCNV) was defined as the mean negative amplitude in the time window 500 ms preceding the imperative stimuli on occipital scalp locations.

### Statistical analysis

A mixed design, factorial, and repeated measures analysis of variance (ANOVA) was used to analyse the behavioural performance by means of RTs, %CRs and Errors data. For RTs and %CRs analysis, the within-subject factors were the condition *validity of the cue *(valid vs. invalid) and *side of presentation *of the target (left vs. right), and the between-subject factor was the subject's *group *(RRMS vs. BMS vs. Controls). In the case of % Errors, we used the same statistical design although *side of presentation *was replaced with *type of error *(misses vs. target error vs. false positive).

For all ERP comparisons, voltage measurements in different electrodes were taken for each ERP component and introduced in the ANOVA. Therefore, *electrode *factors could be represented by frontal, central, parietal, occipital or temporal localizations according to definitions of each ERP component, whereas *hemispheric *factor could be constituted by left, right or midline hemispheric localizations. Moreover the factors, *side of presentation *of the stimuli (left vs. right) and *validity of the cue *(valid vs. invalid) were introduced into the ANOVA as within-subject factors when appropriate. The between-subjects factor was always grouped in three levels (RRMS vs. BMS vs. Controls).

In the case of CNV, separate repeated-measures ANOVAs for each CNV period were performed with a new within-subject factor *cued side *(left vs. right) as well as *electrode *(frontal vs. central vs. parietal vs. occipital) and hemispheric (left vs. midline vs. right hemispheric) factors. For LN wave, the same statistical analyses were conducted for both standard and target stimuli using frontal, central and parietal electrodes, but the *side of presentation *factor was collapsed to maximize effects in order to achieve adequate signal-to-noise ratio.

Since ERP latencies followed a normal distribution, parametric tests were applied. When appropriate, the Geisser-Greenhouse (G-G) procedure was applied to correct degrees of freedom. Post hoc comparisons (*t *tests and Bonferroni test) were performed when ANOVA yielded significant results. A level of *p *≤ 0.05 was accepted as statistically significant.

### Correlations

Behavioural (RTs, %CR and % errors) and psychophysiological data (amplitude and latency) of different ERP components were correlated with clinical variables (EDSS and disease duration) in MS patients using Pearson's and Spearman's correlation coefficients where necessary. Previously, partial correlations adjusted for age were used to screen psychophysiological variables for their relationship with EDSS and disease duration. For amplitude and latency measures, if an ERP component obtained several good correlations in different electrodes, we chose the maximum values for each ERP component.

### Cluster analysis

Cluster analysis was performed in order to identify the natural occurrence of subgroups of patients with similar properties according to their behavioural and psychophysiological characteristics. A 2-stage cluster analysis [32] was used to build up the cluster solution, i.e. hierarchical clustering followed by non-hierarchical clustering.

Initially, the behavioural and psychophysiological data from the MS patients were converted to Z-scores based on the data from the control group in order to ensure equal weighting of different symptoms in the clustering procedure. The variables introduced in the cluster analysis were those which most clearly discriminated between groups in terms of statistical significance (p > 0.035) after performing ANOVA analysis. The first step of this cluster analysis employed a Ward's method of minimum-variance (hierarchical) to explore and to determine the number of clusters and thus to provide the initial solution for the second step. Since outliers might severely distort the results of cluster analysis, in this first step some participants were identified as outliers (those showed highest square Euclidean distances with regard to the others clusters) and removed from the following statistical step. The second step employed a K-means cluster analysis (non-hierarchical) using the nearest neighbour joining method computing Euclidean distances among all participants.

K-means procedure separates subjects into a pre-specified number of cluster groups. The procedure allows some flexibility in terms of choosing different indicator variables and numbers of clusters to be formed. The present study also followed some recommendations [32,33] to validate the proposed clustering solution: partitioning of clustering variables; validating the clustering solution, and profiling the clustering solution with variables other than those in the clustering procedure. Finally, in order to verify whether the clusters were really different from one another, we subsequently performed a new descriptive analysis and an ANOVA with the clusters as between-subjects factors and the new Z-scores as within-subjects. Post-hoc analysis to compare each cluster with the other for every cognitive Z-score of behavioural and psychophysiological measures was carried out taking into account a significant difference of *p *< 0.05.

## Results

### Behavioural data

Table 2 summarizes the behavioural results after application of the Posner task for both control and MS groups. These behavioural data were partially reported in a previous study [28] although new analyses were computed in this study.

**Table 2:** Mean reactions times (RTs), percentage of correct responses (% CRs) and percentage of errors (% Errors) for different groups

			*Groups*		
	
Behavioural		Controls *	RRMS *	BMS	*p*
					
*RTs (ms)*	Valid	433 ± 50	492 ± 47	549 ± 63	-
	Invalid	467 ± 56	510 ± 43	560 ± 45	--
	Total	450 ± 53	501 ± 43	555 ± 51	**<0.001 ^a,b,c,d^**
					
					
*% CRs*	Valid	96 ± 4	94 ± 5	81 ± 23	--
	Invalid	97 ± 5	92 ± 7	79 ± 21	--
	Total	96 ± 5	93 ± 6	80 ± 23	**0.003 **^**a**^
					
					
*% Errors*	Valid	1	3	8	
	Invalid	3	4	12	
	Total	4	7	20	**0.005 **^**a,b,c**^
					

***a ***= between control and MS groups but not differences between both MS groups;

***b ***= between RRMS and BMS groups, after post-hoc analysis;

***c ***= between control and BMS groups, after post-hoc analysis;

***d ***= between control and RRMS groups, after post-hoc analysis;

* = Groups who showed statistical differences for validity of the cue (invalid > valid)

MS groups obtained a worse performance than the control group with higher RTs (F (2,42) = 14.947, *p *< 0.001) and a lower CRs percentage (F (2,42) = 6.790, *p *= 0.003). Between MS groups, BMS patients showed poorer results than RRMS patients as well as a lack of *validity effect *(RTs invalid minus RTs valid condition).

Furthermore, the analysis of Errors percentage (*missed*, *target error *and *false positive*), after partial-weight correction between valid and invalid trials, revealed that there was a significant *error x group *effect (F (4,86) = 5.780, *p *= 0.005) caused by the fact that MS patients obtained a higher amount of errors. Post-hoc tests proved that there was a significantly higher error rate in the BMS group in comparison with the other two groups for *missed*-error type (RRMS: *p *= 0.040; Controls, *p *= 0.005), which largely represents the lack of accuracy in the benign patients. In general, and for all groups during the task performance, the most important error type was the *missed*-error for invalid trials (*validity of the cue *x *type of error *effect: (F (2,86) = 11.279, *p *< 0.001), whereas the *false positive*-error type was practically absent in all groups.

### Event-related potentials

**Figure 2:**
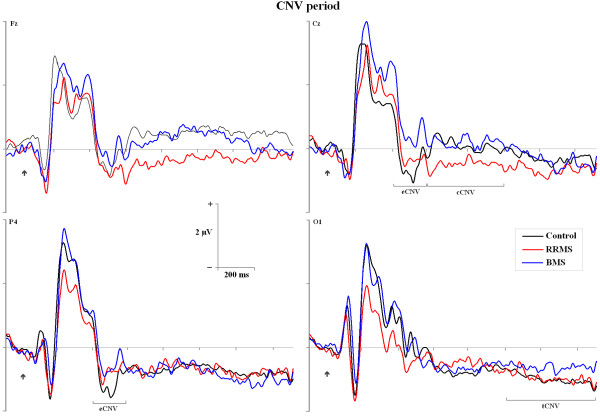
**Event-Related Potentials (ERP) elicited by cues and the subsequent CNV period for the three groups in different positions of the scalp**. The black arrow indicates when the cue stimulus is presented and the black arrow with an asterisk when the imperative stimulus is presented. The marked black lines represent: eCNV, cCNV and tCNV periods.

**Figure 3:**
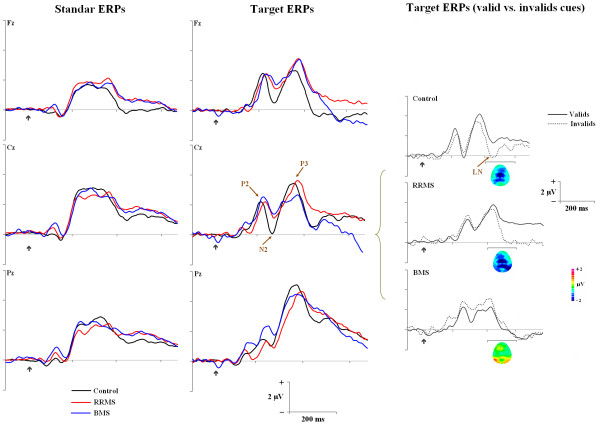
**Event-Related Potentials for different groups associated to: a) standard stimuli, b) target stimuli, and c) valid and invalid cues for target stimuli period for three groups in different scalp positions**. In the right column the period (black lines) where the LN was analyzed and the voltage maps for *difference *waveform are showed. The black arrow demonstrates when the imperative stimulus is presented.

The CNV waveforms are shown in Figure 2 and the grand-mean ERPs elicited by the visual standard and target stimuli and the difference waveforms are displayed in Figure 3. After the cue stimulus, three CNV periods were clearly identified on the grand average, whereas with the arrival of standard or target stimuli, typical ERP components were developed. The standard stimuli elicited an early inflection previous to the P1 component which we termed as frontal eN1 (mean latency = 120 ± 9 ms), P1 component (mean = 133 ± 5 ms), N1 component (mean = 174 ± 11 ms) and P3 component (mean = 352 ± 12 ms) components. On the other hand, the target stimuli elicited a frontal and central P2 component (mean = 222 ± 10 ms), N2 component (mean = 257 ± 11 ms) and P3 component (mean = 368 ± 19) components. Moreover, both standard (mean = 410 ± 21 ms) and target (mean = 443 ± 19 ms) stimuli elicited a negative late negativity (LN) component over midline after the P3 component.

The new factors and levels introduced in the statistical analysis in comparison with that of our preliminary study using only standard stimuli and some ERPs components [29] did not show any new *group *effects for this type of stimuli.

#### CNV period and ERP Amplitude

Table 3 summarizes some results related to amplitude. In relation to the CNV period, a typical increased negative deflection was shown after cue stimulus. The landscape of both periods was highly similar for eCNV, cCNV and tCNV periods between control and MS groups. However, there was a significant interaction *group *x *hemispheric *x *electrodes *(F (2,42)= 2.962, *p *= 0.035) for eCNV. Post-hoc analyses revealed that BMS group had a smaller amplitude (i.e., most positive-going) in comparison with the control group for central electrodes of (*p *= 0.031) and parietal electrodes of right hemispheric (*p *= 0.045). Post-hoc analyses also showed that there were no differences between RRMS and controls, respectively, or between the two MS groups.

**Table 3:** Amplitude and latency of ERP components for target and standard stimuli and for the CNV periods

			*Amplitude*					*Latency*		
				
										
		Controls	RRMS	BMS	*p*		Controls	RRMS	BMS	*p*
***ERPs***		*Mean ± SD*				***ERPs***	*Mean ± SD*			
										
										
(○) eN1					**0.001 ^a^**					
	Valid	-0.85 ± 2.4	0.47 ± 1.2	0.97 ± 1.2						
	Invalid	-0.63 ± 0.9	0.33 ± 1.5	0.96 ± 2.1						
										
(○) P1					**0.020 ^a,b,c^**	(○) P1				0.558
	Valid	1.59 ± 1.8	1.86 ± 1.3	1.59 ± 1.0			132 ± 6	135 ± 6	136 ± 7	
	Invalid	1.17 ± 1.5	1.19 ± 1.2	2.09 ± 2.1			133 ± 7	133 ± 5	133 ± 6	
										
(○) N1					0.419	(○) N1				**< 0.001 ^a,c^**
	Valid	- 0.95 ± 1.2	- 1.41 ± 1.1	- 1.01 ± 1.7			174 ± 11	179 ± 10	188 ± 8	
	Invalid	-0.98 ± 1.5	-1.46 ± 1.1	-0.81 ± 1.5			173 ± 11	181 ± 10	188 ± 9	
										
(○) P3					0.294	(○) P3				**0.003 ^a,b,c,d^**
	Valid	4.99 ± 2.4	4.38 ± 4.8	4.56 ± 2.7			352 ± 1	371 ± 10	381 ± 18	
	Invalid	5.67 ± 1.4	5.18 ± 1.5	4.61 ± 2.5			353 ± 13	375 ± 17	390 ± 12	
										
(⊙) P2					0.232	(⊙) P2				**< 0.001 ^a^**
	Valid	5.40 ± 2.8	5.37 ± 3.3	5.26 ± 3.7			219 ± 12	240 ± 13	241 ± 17	
	Invalid	5.99 ± 3.7	6.25 ± 2.7	7.25 ± 1.5			226 ± 11	242 ± 12	249 ± 13	
										
(⊙) N2					0.169	(⊙) N2				**0.002 ^a^**
	Valid	-0.30 ± 1.7	-0.16 ± 1.8	-0.52 ± 3.8			252 ± 13	268 ± 10	271 ± 17	
	Invalid	-0.10 ± 2.6	-0.81 ± 2.4	-0.40 ± 3.1			262 ± 11	270 ± 12	269 ± 12	
										
(⊙) P3					0.239	(⊙) P3				**< 0.001 ^a^**
	Valid	8.95 ± 3.7	7.63 ± 4.3	7.13 ± 4.4			372 ± 19	397 ± 15	399 ± 20	
	Invalid	9.81 ± 3.9	8.36 ± 4.1	9.22 ± 4.2			363 ± 20	381 ± 20	390 ± 17	
										
										
(○) LN		-1.72 ± 1.1	-1.80 ± 1.5	-1.68 ± 1.5	0.718					
(⊙) LN		-2.23 ± 2.1	-3.24 ± 2.0	-1.13 ± 1.5	**0.031 ^b^**					
										
										
										
eCNV		-1.82 ± 1.7	-0.87 ± 1.1	-0.18 ± 2.2	**0.035 ^a, c^**					
cCNV		1.13 ± 0.8	-0.57 ± 0.8	0.89 ± 1.0	**0.043 ^a, d^**					
tCNV		0.57 ± 0.6	0.96 ± 0.7	0.74 ± 0.9	0.632					
										

Means and standard deviations (SD) of electrodes and regions of interest used for each ERP-component ANOVA analysis are shown. P values show main statistical effects involving the group factor.

[⊙ = target stimuli; ○ = standard stimuli].

***a ***= *p *< 0.05 between control and both MS groups;

***b ***= *p *< 0.05 between RRMS and BMS groups;

***c ***= *p *= < 0.05 between control and BMS groups;

**d **= *p *< 0.05 between control and RRMS groups.

Regarding cCNV period, there was a main effect of *group *(F (2,42) = 3.385, p = 0.043), showing that RRMS patients had a significant increase (more negative) of mean cCNV amplitudes on frontal electrodes in contrast to the control group (*p *= 0.045). However, there were no significant differences in relation to tCNV amplitudes between different groups. There was a main *electrode *effect (F (2,42) = 12.589, *p *< 0.001), indicating that the component showed typical modulations and it was significantly higher (i.e. most negative-going) in posterior sites than anterior sites of the scalp.

Visual inspection of grand-average waves suggested that amplitude of ERP components to standard stimuli (P1, N1 and P3) and to target stimuli (P2, N2 and P3) were fairly well preserved in both groups of MS patients. However, this did not occur for the frontal eN1 component elicited by standard stimuli, which had a more negative amplitude in the control group, whereas a positive deflection appeared for the MS groups. Statistical analysis confirmed a significant main *group *effect on this component on middle line and right hemispheric over frontal regions for controls compared with MS patients (F (4,84) = 11.303, *p *< 0.001), whereas there was no differences between MS groups.

Although the grand means data of P1 showed typical modulations of attention effect with Posner tasks (enhanced amplitude of P1 for valid and attended condition in comparison with invalid and not attended condition), this effect was not statistically confirmed (F (1,42) = 1.523, *p *< 0.224). There was an interaction effect of *group *x *validity of the cue *on the P1 amplitude (F (2,42) = 4.295, *p *= 0.020). Control and RRMS groups showed higher amplitudes for valid trials compared to invalid trials, which was not observed in the BMS group who showed the highest amplitude of all groups for invalid trials, however, post-hoc comparisons did not confirm this group effect.

Both N1 and P2 components did not show group effects for amplitude. However, the P2 component showed in all groups, that stimuli preceded by invalid trials had higher amplitude respect to stimuli preceded by valid trials (*validity of the cue *effect: (F (1,42) = 12.847, *p *< 0.001) and over frontal electrodes in comparison with central electrodes (*electrode *x *hemispheric *effect: (F (2,84) = 3.536, *p *= 0.034). Equally, although the control group clearly exhibited a larger average of N2 amplitude than both MS groups for target stimuli (mean amplitude = Controls: -0.41 ± 3.5 μV; RRMS: 1.39 ± 3.7 μV; BMS: 2.18 ± 4.4 μV), this was not statistically significant (F (2,42) = 1.857, *p *= 0.169), probably caused by a high intersubject variability in all groups. In general, a higher negative amplitude was observed in central regions in comparison with frontal regions (*electrode *effect: F (1,42) = 14.743, p < 0.001), and for right hemispheric compared to left hemispheric positions (*hemispheric *effect: (F (2,84) = 3.814, *p *= 0.028).

Significant between-group differences in the amplitude P3 component were not observed in either the standard or target stimuli. All groups obtained higher amplitudes for invalid vs. valid trials for target stimuli in the amplitude P3 component with maximum effect in parietal regions of mean line in comparison with other scalp positions analyzed (F (6,252) = 2.734, *p *= 0.034). However, for standard stimuli, and in spite of the BMS group not obtaining higher amplitudes for invalid vs. valid trials with standard stimulation (in comparison to RMSS or control groups), statistical differences were not found with respect to the validity of the cue between groups for the amplitude of the P3 component.

The LN amplitude after subtraction (invalid minus valid) showed a central-parietal dominant LN at latency of 400-500 ms (mean = 447 ± 19 ms) in all groups which was also clearly higher to target stimuli compared with standard stimuli (F (5,210) = 4.217, *p *= 0.006). Only ANOVA for target stimuli of the LN component revealed a significant *group *effect (F (2,42) = 3.773, *p *= 0.031) caused by the fact that, over the central midline, the LN component was larger in amplitude (most negative-going) in the RRMS group than in the BMS group (*p *= 0.028) but there were no differences compared with the control group (*p *= 0.399). Finally, LN amplitude showed that, both for standard (*hemispheric *effect: (F (2,84) = 6.321, *p *= 0.007) and target stimuli (*hemispheric *x *electrode *effect: (F (4,168) = 3.446, *p *= 0.024), central regions had higher amplitude with respect to frontal or parietal regions.

#### ERP Latency

Table 3 summarizes the results related to latency. The ERPs to standard stimuli were P1, N1 and P3 components. The ANOVA on the latency of the standard-evoked P1 revealed that there was no effect of *group *between different groups (F (2,42) = 0.591, *p *= 0.558) and that there was a significant interaction *side of presentation *x *hemispheric *x *electrode *(F (2,84) = 12.226, *p *< 0.001) caused by the fact that faster latencies are obtained over contralateral regions of the side of presentation of the stimuli as compared to ipsilateral regions, and also because the P1 latency is contralaterally faster on the right (vs. left) side of stimulus presentation and over temporal and occipital (vs. parietal) areas. However, N1 component showed a *group *x *electrode *interaction effect (F (4,84) = 8.927, *p *< 0.001), indicating post-hoc comparisons that BMS patients had a larger N1 latency than the control group over parietal (*p *< 0.001) and occipital (*p *< 0.001) sites. RRMS patients did not show differences in N1 latency compared with controls or BMS patients.

With respect to P3 latency of standard stimuli, there was also a statistically significant interaction *group *x *electrode *(F (6,126) = 5.149, *p *< 0.003), indicating that the P3 latency of the control group was faster than both MS groups (*p *< 0.001 respectively) over all scalp sites analyzed, whereas RRMS had a faster P3 latency than the BMS group only over central (*p *= 0.033) and parietal (*p *= 0.015) areas. Despite the invalid trials (vs. valid trials) showing significantly longer P3 latencies both for controls and MS patients, this was not confirmed statistically.

ERP to target stimuli consisted of P2, N2 and P3 components. P2, N2 and P3 latencies showed a strong main *group *effect between controls and MS patients, but not between MS groups. Therefore, both MS groups had equally affected P2, N2 and P3 latencies. Thus, controls were faster for P2 latency (F (2,42) = 18.246, *p *< 0.001), N2 latency (F (2,42) = 6.987; *p *= 0.002) and P3 latency (F (2,42) = 10.153, *p *< 0.001) than both MS groups, although post-hoc analysis did not show differences between RRMS and BMS groups.

In general, invalid trials of target-ERP components obtained longer latencies in all groups. There was also a significant interaction *hemispheric *x *validity of the cue *x *group *on the P3 latency (F (4,84) = 2.688, *p *= 0.047), whose post-hoc *t*-tests indicated that the Control group had shorter latencies compared with the RRMS group to valid stimuli over midline (*p *< 0.001) and left hemispheric (*p *= 0.001) sites, whereas the control group obtained shorter latencies compared with the BMS group to invalid stimuli over midline (*p *< 0.001) and right hemispheric (*p *= 0.005) sites of the scalp.

Since there were few trials with target stimuli and probably reduced noise-signal, P1 and N1 components were not analyzed. However, there was no group effect on the latency of LN that appeared following the P3 component for both standard and target stimuli.

### Correlations

The MS clinical variables were correlated with behavioural and ERP data. Our results showed that a statistically significant correlation was not observed between EDSS scores and any behavioural or psychophysiological measure. Despite several apparently strong correlations between disease duration and latencies in different ERP components (e.g., between disease duration and P3: *r *= 0.790, *p *= 0.001), most of these correlations did not remain significant after partial correlations were adjusted for age. P2 (*r *= 0.506, *p *= 0.008) and N1 (*r *= 0.500, *p *= 0.009) latencies were the only measures that maintained moderate correlations after being adjusted for age.

### Cluster analysis

The first step of the cluster analysis suggested three to four clusters to explain the present sample, after removing outliers. Initially, a three cluster solution appeared most suitable (as controls fell into an intact cluster and all MS patients fell into two separate clusters), however, a four cluster solution was selected as healthy participants strongly tended to split into two different clusters, practically without affecting the patient clusters from the three cluster solution. This approach allowed exploration of the characteristics that divided the control group.

Therefore, a four-cluster solution was chosen as the optimal solution with the outlier subjects removed from analysis after an examination of Euclidean distances between cluster centres, the between-cluster mean squares, and the within-cluster mean squares, and adopting the suggested procedures for validating the clustering solutions for K-means cluster analysis [32,33]. In addition, subsequent ANOVAs and post-hoc analyses (with the clusters as between-subject factor and Z-scores as within-subjects factors) revealed that the differences were highly significant for the included parameters.

**Figure 4:**
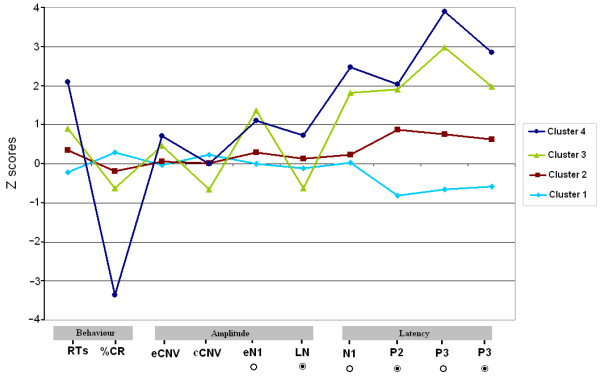
**Profiles of psychophysiological findings for the four clusters**. [⊙ = target stimuli; ○ = standard stimuli] Collectively, these findings suggest that the relapsing-remitting course of MS appears to form a more homogenous group of patients in this disease while the benign course of MS forms a more heterogeneous group in terms of altered cognitive processing. Thus, RRMS patients appear to have a relatively preserved behavioural performance with mild/moderate abnormalities of early-latency ERP components and moderate/severe impairment for P3 latency. However, our findings also show that some RRMS patients may develop important cognitive disturbances during typical relapsing-remitting stages of the disease. On the other hand, BMS patients tended to demonstrate a high heterogeneity according to their psychophysiological abnormalities. Thus, BMS patients clustered into Cluster 2 without signs of cognitive impairment and into Cluster 3 with moderate cognitive disturbances. Furthermore, BMS patients even fell into outlier cases with more severe abnormalities.

The profiles of the four groups are summarized in Figure 4 and Table 4. Cluster 1 took up 50% of the controls only, whereas Cluster 2 accounted for 50% of controls, 11.8% of RRMS and 10% of the BMS patients. Cluster 3 and Cluster 4 only consisted of MS patients. Thus, Cluster 3 accounted for 68% of the total RRMS patients' sample of our study as well as 30% of BMS patients, whereas Cluster 4 accounted for 17.4% of RRMS patients and 40% of BMS patients.

**Table 4:** Values represent mean standardized scores for cluster centers after computing k-means cluster analysis

	Cluster 1	Cluster 2	Cluster 3	Cluster 4	Outliers		
	CONTROLS = 50%	CONTROLS = 50% RRMS = 11.8% BMS = 10%	RRMS = 68% BMS = 30%	RRMS = 17.4% BMS = 20%	BMS = 20%	*p*	*post-hoc*

***Demographics***							
Age	28.4 ± 5.7	36.0 ± 9.7	40.7 ± 9.4	38.8 ± 4.1	56 ± 0.0		
Dis. duration		5 ± 6.1	6.2 ± 4.6	7.4 ± 3.8	14.1 ± 3.5		
EDSS		1.8 ± 1.44	2.1 ± 0.8	1.8 ± 4.7	2.7 ± 0.3		
							
							
***Behavioural***							
RTs	-0.222	0.344	0.908	2,094		**<0,001**	b c e f
%CRs	0.287	-0.191	-0.632	-3,366		**<0,001**	c e f
							
							
***ERP Latency***							
(○) N1 _P4_	0.024	0.236	1,817	2,475		**<0,001**	b c d e
(⊙) P2 _Fz_	-0.817	0.877	1,736	2,696		**<0,001**	a b c d e f
(○) P3 _Fz_	-0.556	0,478	2,096	2,921		**<0,001**	a b c d e
(○) P3 _Cz_	-0.652	0.763	2,988	3,897		**<0,001**	a b c d e f
(○) P3 _Pz_	-0.734	0.869	3,133	4,056		**<0,001**	a b c d e f
(⊙) P3 _Fz_	-0.438	0.466	0,863	2,092		**<0,001**	a b c e f
(⊙) P3 _Cz_	-0.579	0.624	1,373	2,350		**<0,001**	a b c e f
(⊙) P3 _Pz_	-0.607	0.582	1,054	2,041		**<0,001**	a b c e
							
							
***ERP amplitude***							
							
eCNV_Cz_							
eCNV_P4_	-0.034	0.052	0,466	0,909		**0,038**	c
cCNV_Fz_	-0.186	0.063	0,850	-0,221		**0,047**	c
(○) eN1_Fz_	0.232	0.019	-0,652	-0,221			d
							
(⊙) N2_Cz_	0.002	0.293	1,370	1,109		**0,002**	b c d
(⊙) LN_Cz_	-0.331	0.043	-0,758	0,368		0,054	-
(⊙) LN _C4_	-0.122	0.138	-0,626	0,726		**0,018**	c d f
	-0.026	0'066	-0,398	-0,028		0,639	-

Significant *p *values of a new cluster-related ANOVA (using Z-scores as within-subject factors and the different clusters as between-subject factor) are also displayed. The percentage of cases and the contribution of each different experimental group to cluster solutions are also shown. Outlier cases are shown but they were not included in the new ANOVA. In general, ERP latencies had a great weight on splitting the clusters than ERP amplitudes. New demographic values (mean and SD) for age, disease duration and EDSS scale are also shown for each cluster. Differences between clusters for demographic values were not found. Different statistical differences were found between clusters as follows:

[⊙ = target stimuli; ○ = standard stimuli].

***a ***= *p *< 0.05 between Cluster 1 and 2; ***b ***= *p *< 0.05 between Cluster 1 and 3;

***c ***= *p *< 0.05 between Cluster 1 and 4; ***d ***= *p *< 0.05 between Cluster 2 and 3;

***e ***= *p *< 0.05 between Cluster 2 and 4; ***f ***= *p *< 0.05 between Cluster 3 and 4.

Significant differences were found among Cluster 2 and Cluster 1 (over 1.5 or 2 standard deviations) in terms of latency for P2 (*p *< 0.001) and P3 (*p *< 0.001). Cluster 1 was different (*p *> 0.001) from Cluster 3 for all latency-scores as well as RT score (*p *= 0.024) and for early frontal N1 (*p *= 0.005), eCNV (*p *= 0.019) and cCNV (*p *= 0.039) amplitude-scores. Moreover, Cluster 1 was also different from cluster 4 for all scores measured. Cluster 2 was similar to Cluster 3 in terms of behavioural performance, although they were different for latency-scores of P3 (*p *< 0.001) and of P2 (*p *= 0.009), as well as for amplitude-scores of frontal eN1 (*p *= 0.038) and LN (*p *= 0.048). Cluster 3 was different from Cluster 4 for all behavioural and latency scores and for LN amplitude (*p *= 0.012) but not for eCNV and frontal eN1 amplitude-scores.

Cluster 1 seemed to include the younger subjects of the control group (28.4 ± 5.7) than those in Cluster 2 (36.0 ± 9.7), although it was not statistically significant. Clusters accounting for MS patients were not different in terms of age, disease duration or EDSS score. In any case, it was observed that clusters consisting of patients with a worse psychophysiological performance also had a longer disease course. EDSS scores did not show differences between clusters.

It is also important to highlight that outliers' cases account for 20% of benign patients who were characterized by longer disease duration, a very poor behavioural performance and an abnormal psychophysiological pattern in terms of several standard deviations in comparison with other clusters.

## Discussion

In the present study we used an attentional visual-spatial task with central cues, the so-called central cue Posner's paradigm, in order to investigate the altered cognitive processing in two subtypes of MS patients and controls. At the same time we explored the nature and pattern of these cognitive abnormalities and their relationship with clinical variables using a multivariate statistical approach.

### Behavioural performance

MS patients demonstrated a slower response speed than controls when detecting the mere presence of a stimulus previously cued (attentional orienting) as well as when they had to detect a stimulus which appeared over the opposite cued site (attentional reorienting).

Our results replicate other studies in healthy subjects, which have observed an increase of RT for an invalid cueing condition compared with a valid cueing condition [25,26,34]. Orienting to cues provides information about where the target will occur, with consequent benefits for RT. It is expected that the processing of a target stimulus that is not previously cued, takes longer, or causes more errors, than that of a cued target stimulus. Control and RRMS groups showed this "valid effect", but patients of the BMS group did not.

Results obtained by BMS patients cannot be easily attributed to a deficit in the disengaging of attention, since these patients obtained a similar RT in valid and invalid condition. Their performance could be attributed rather to a deficit in engaging attention or follow-up of cue, since they seem to deal with all stimuli equally. Similarly, these differences cannot be explained by a speed-accuracy trade-off because MS groups were also less accurate than the control group. Both RRMS and BMS groups had significantly lower CR percentage and more errors than the control group, with predominance of the *missed *type. In patients with BMS, the *missed *type error makes up the vast majority of lack of accuracy during the task. Since we have considered an error type *missed *when no response to the target stimuli occurred, or if the response occurred 700 ms after target stimuli, then the lack of accuracy of patients with BMS could be interpreted as a slowed cognitive processing on attentional tasks.

Slower reaction times in MS patients have been frequently reported [35,36] as well as frequent attentional deficits [37,38], and a slowing of mental processing independent of motor slowing has been suggested [37,39,40]. This slowing of the speed of mental processing and other cognitive dysfunctions can already appear early in the natural history of the disease, or even in clinically isolated syndromes suggestive of MS [41,42]. It can also be predictive of global cognitive decline [37] and is particularly pronounced with visual and auditory tasks [37,40,43].

The findings of our study suggest that subtypes of MS disease may be associated with a moderate/severe attentional impairment and with information processing deficits. Compared to RRMS patients, BMS patients were slower and less accurate, suggesting that BMS patients could incur greater information processing impairments. These deficits could be affecting attentional functions in terms of attentional orienting and reorienting affecting the BMS disease subtype more than other subtypes of MS (such as RRMS) despite an apparent milder physical disability.

Although our results have clearly shown that BMS patients were slower and had less accuracy in visually presented information processing than RRMS patients, there are very few studies (most of them very recent) that have explored or found evidence of altered cognitive processing in the benign subtype of MS [9,10,44]. Furthermore, very few functional studies have tried to assess different aspects of attentional processes and information processing in MS patients with a benign profile, using cognitive paradigms like the Posner paradigm [28] or the Stroop paradigm [45].

Both RRMS and BMS patients may use different cognitive strategies in task performance, although this suggestion was not checked. For instance, some studies have demonstrated that when patients with MS are provided with additional time to process information, they perform as accurately as controls [36].

### Event Related Potentials

The analyses of the ERP data also revealed some interesting findings. In combination with these behavioural data, electrophysiological results allow us to propose the existence of distinct levels of altered stimulus processing in MS patients, notably in attentional mechanisms and different stages of information processing.

#### CNV period and ERP Amplitude

The topographical distribution of eCNV clearly separates BMS patients from controls, but not from RRMS patients. When regions are considered separately, eCNV is smaller (amplitudes more positive-going) in BMS patients than in controls at central and parietal but not frontal sites. On the other hand, a striking finding of the present study has been the more negative amplitude of cCNV period at fronto-central sites that RRMS patients showed in comparison to the control group but not the BMS patients.

According to the functional hypothesis of early period of CNV [15,46,47] current results may indicate a reduced or worse activation of orientation and preparation mechanisms in BMS patients, after the presence of a cue. RRMS patients, however, might be exhibiting an increased attention at the beginning of the preparation stage or greater task motivation since the amplitude of CNV increases (more negative) with the force required to make a response, as well as when more attention is directed to the response [48,49]. These amplitude abnormalities found in different temporal periods of CNV both for BMS and RRMS patients may therefore be revealing diverse neural mechanisms involved during preparation performance, which may be affected in different degrees. However, both MS groups appear to have the amplitude of the final period of CNV intact, which is traditionally related to sensory preparation for the imperative stimulus as well as to preparation for the motor act.

Consistent with a large body of evidence, the CNV is a sustained slow potential that develops during the interval between two task-relevant stimuli, with the second stimulus usually requiring a motor response [50,51]. After the initial response to the warning stimulus is completed, an early negativity or eCNV develops over prefrontal, precentral or parietal areas depending on the task or paradigm used [52,53]. Specifically, a change on eCNV amplitude has been observed when a spatial cue is presented in comparison to when the cue is not presented. This finding has been functionally interpreted in terms of activation of an executive mechanism controlling orientation or attention to a stimulus, assigning specific neural resources to the cued side and the posterior tCNV and suitable preparation responses [46,52,53]. In general, the main contributor to the CNV is the activation of fronto-parietal networks indicating the endogenous attentional effort during the CNV period, and the activation of the task-related neural set [15,16,47,54].

Regarding BMS patients, our findings related to CNV seem to demonstrate that BMS patients, with the slow advance of disease, could in turn develop deficits in the fronto-parietal networks involved in tasks with cued stimuli. When adopting the view that early CNV activity (starting around 500 ms after cue onset) is indicative of the start of cue orientation processes, whereas the later CNV wave reflects expectation of the response and target location, it could be speculated that BMS patients invest less effort or have a compromised attentional mechanism in cue-orientation processes. Some of the previously discussed cortical alterations found in MS patients, which are linked to neural generators of CNV, have been partly reported in recent studies with BMS patients [9,10,45] and could be the reason that BMS patients showed cognitive abnormalities beyond the behavioural slowing that we have highlighted.

RRMS patients, however, appear to have good preservation of the brain mechanisms indexed by the CNV, showing even greater amplitude during the specific time period of CNV. Previous studies have shown that the CNV amplitude may be more negative when more attention is focused on the task [46,48] and it is jointly determined by the two processes of attention and arousal [48]. Therefore, RRMS patients could be mobilizing a greater amount of neural resources during initial periods of CNV (around mean 800 ms after cue onset) related to expectation and preparation for cognitive and behavioural responses.

Furthermore, this finding may also be connected to other striking results related to the higher amplitude of the LN component that RRMS patients obtained in comparison to BMS patients. However, any interpretation regarding this finding should be cautious since the only differences found were between the patient groups but not between patients and controls. Neuroimaging and electrophysiology studies have clarified some aspects related to the usual neural sources for the LN wave and other late ERP negativities during the performance of different conflict task, including spatial conflict [55-57], further indicating that the size of conflict is important in the generation of associated cortical activity.

A first interpretation for the largest amplitude of LN and cCNV in RRMS group could refer to an increase in cortical activity and neural resources associated to higher-order processing of stimuli. In this respect, the amplitude of long-latency of ERP components is considered as an index of brain activity that is required during a task, or proportional to the amount of attentional resources devoted to a given task [58-60]. Consequently, the higher LN amplitude for RRMS (and the lower amplitude for BMS) as well as higher cCNV, could also suggest the existence of compensatory cortical activation and brain reorganization when these patients perform attentional or memory tasks, in accordance with some functional neuroimaging findings during performance of the spatial tasks [61-64]. RRMS patients, therefore, could be manifesting a particular brain organization or increasing amounts of neural sources (indexed to higher ERP amplitudes) involved in specific time periods which relate to preparation performance and spatial conflict monitoring in the Posner paradigm.

However, some limitations of our study do not allow us to conclude this point of view. As far as we know, there are no other studies that have previously assessed the functional mean of CNV in MS, so we cannot compare our results in MS patients with other studies. Nevertheless, further studies should aim to explore and verify these aspects.

Regarding amplitudes of other ERP components analyzed in our study, we tried to find the electrophysiological correlates of the behavioural advantage of validly cued targets. Typically, the P1 amplitude is enhanced to valid cues. However, the control and the RRMS groups showed greater amplitude to the valid condition compared with BMS patients, suggesting that BMS patients seem to have a lack of validity effect to amplitude P1 [28]. Some improvements, such as increasing the size of our sample, might be made in future studies to determine better electrophysiological correlates of behavioural valid effect.

In contrast to our current results, some authors have reported frequent decrease of ERP component amplitude in MS patients, but in particular for N2 and P3 waves and for the auditory modality. A decrease of amplitude for P3 [65,66], for P2 and for P3 [18], for N2 and for P3 [67], for *mismatch negativity *complex and P3 [68], or for P2 and N2 waves [19,69,70] has been reported in MS patients. These results have been interpreted in terms of information processing difficulties and an impairment on attentional orienting mechanisms related with a disruption of the cortical-cortical or cortical-subcortical neural connections which occurs as a consequence of demyelination and axonal degeneration.

The reason for this apparent discrepancy with respect to our amplitude-related findings could be due to the fact that we included MS patients with a lower EDSS in our study. Amplitude alterations seem to be more pronounced in cognitively impaired patients or when patients with high EDSS or secondary-progressive forms are included [18,70]. It is commonly accepted, that in advanced courses of MS and in patients with a high disability, the decrease of ERP amplitude could be a consequence of widespread demyelination and axonal degeneration in the brain of these patients, causing disruption of cortical-subcortical network connections and their recruitment for neural activity [22,23]. However, despite conservation of amplitude for standard ERP components, the use of more refined cognitive paradigms like the Posner paradigm could detect more ERP abnormalities related to silent cognitive disturbances in these types of MS patients [24]. Thus, similar preservation of amplitude has been reported for traditional ERPs components, but specific ERP components associated to working memory paradigms showed a reduced amplitude [41,71,72].

### ERP Latency

Exploring the ERP latency results of our study allowed us to observe that MS patients demonstrated an increased latency in most ERP components analyzed. Thus, our results showed an increased of P2, N2 and P3 latencies in both MS groups respect to healthy subjects. Moreover, BMS patients obtained an increase of latency for N1 and P3 components in comparison with RRMS patients when standard stimuli were analysed (which involved a higher number of stimuli than target condition). Consistent with our findings on amplitude or behavioural measures, these differences seem to confirm a greater alteration in cognitive processing for MS patients with a benign profile. Increased ERP latencies in BMS as well as RRMS led us to suggest that ERPs may indicate subtle degrees of cognitive dysfunction in different courses of MS disease.

Thus, in all MS patients of our study, latencies of the ERP components P2, N2, and P3 were longer than in healthy subjects, which is quite consistent with the results of previous studies. Most studies using an ERP approach in MS have reported abnormalities of the latency of ERP components, particularly for P3, both in auditory modalities [17-21,66-68] and visual modalities [28,70] or using both modalities [65,73,74]. Some of these studies have also reported an increase in latencies or a decrease in the amplitude for N1 component [18,28], P2 component [17-19,21,67,69,70] or N2 component [18-20,67,69,70]. Similarly, a longer latency for more specific ERP components in working memory paradigms and similar tasks has been found [41,70,71].

In this context, these discrepancies might result from differences in the task or the degree of cognitive abnormalities in the studied patients. Thus, when results are divided in terms of different subtypes of MS, patients with a chronic stage of disease or higher neuropsychological impairment obtain more important abnormalities on ERP latencies [18,21,41,65].

The current study is highly compatible with the idea that abnormalities in long-latency ERP components are not simply due to a different processing speed in sensory visual pathways, but the consequence of central nervous system demyelination. In general, the great majority of findings related to ERP latency abnormalities [23,24] have been interpreted, either in terms of a consequence of demyelinating lesions in primary afferent pathways resulting in prolonged late ERP components [17,18,21], or as a functional disconnection of subcortical regions from higher cortical areas due to demyelinating plaques [22,73,74].

### Cluster analysis

The main aim of this analysis was to determine whether there would be subtypes of MS patients based on different combinations of their cognitive profile and psychophysiological pattern, and to investigate which parameters have a greater weight to split these participants.

Taking into account the measured psychophysiological parameters of our study after performing a Posner-like task, we may conclude that from the MS patients who participated in our study, three distinct subgroups were identified (typical relapsing-remitting course and benign profile course): a "non-cognitive deficit" cluster grouped with controls, a "mild cognitive deficit" cluster with some psychophysiological abnormalities, and a "severe cognitive deficit" cluster with widespread cognitive dysfunction. Furthermore, cluster analysis revealed two more interesting findings: firstly, two control groups could be created based on their younger age as well as short ERP latencies, and secondly, some BMS patients could not be clustered into any group (outliers patients) due to their significant cognitive deficit.

Cluster 1 comprising 50% of controls was characterized by a good behavioural and psychophysiological performance with a mean Z-score lower than the other clusters in all measured domains. Participants of Cluster 2 accounted for the rest of healthy controls as well as approximately 20% of RRMS and 10% BMS patients. They were also characterized by a good behavioural performance and normal psychophysiological measures. Between these two clusters, compromising all controls and very few MS patients, the difference demonstrated by the cluster analysis solution was between the latency of P2 and P3 components, which was very close to 1.5 or 2 standard deviations from Cluster 1 in these parameters. This discrepancy between both clusters that took up all healthy subjects may be due mainly to the fact that healthy participants of Cluster 1 were relatively younger than controls of Cluster 2, although it was not statically confirmed. One hypothesis offering a psychophysiological explanation is that of age-related effects on the latencies of ERP components. There is strong evidence that P3 latency (and less so for P2) appears to be affected by age. On this basis, our findings are in line with other authors who have reported a generic increase in long-latency ERP components in controls with age [75,76].

Most MS patients fell into the other two remaining clusters. Thus, a majority of RRMS patients were included in Cluster 3 (70% of our RRMS sample), while Cluster 4 included a proportional clustering of RRMS and BMS patients. Cluster 3 had a normal Posner task performance in terms of CRs percentage and delay in RTs, although they were also characterized by a moderate-high increase of latency-scores in analyzed ERPs (N1, P2 and P3) with respect to Cluster 1 and 2, showing a mild delay in information processing and a non-standard increase of neural activity in high-order process (LN component) associated with periods after the P3 component.

However, Cluster 4 demonstrated a global and severely altered cognitive processing, characterized by mean performances in the range of two or more standard deviations outside those of the control clusters, in all scores measured. They were characterized by a general cognitive delay, both from a behavioural perspective, with high RTs in their responses and an important increase in numbers of errors (*missed*), as a widespread slowing in information processing and abnormalities in high-order processing.

Finally, a specific analysis for outlier cases removed from the clustering procedure revealed that these outliers only belonged to BMS patients and they showed severe impairment in terms of behavioural performance (RTs and %CRs) and psychophysiological parameters (latencies and amplitude) in the range of four or more standard deviations from the controls. For a more detailed description of cluster features, please see the footnote in Figure 4.

Cluster analysis studies on ERP data are extremely scarce, particularly for MS patients. To our knowledge only one study has been published using cluster analysis to describe cognitive subgroups in MS, but with a neuropsychological approach [77]. Consistent with the findings of this study, attentional and information processing deficits may be reliable, early markers that could be used to differentiate the unaffected from the mildly cognitively impaired RRMS patients.

On the other hand, there seem to be some variables that are more relevant than others for clustering the participants of our study. The new ANOVA performed with the clusters as between-subjects factors and psychophysiological scores as within-subjects factors revealed that, in general, latency parameters (N1, P2 and particularly P3 components) were the strongest scores for clustering patients. However, amplitude parameters, probably due to the higher variability within them, seem to be sensitive only to cluster splits formed mostly by patients.

The behavioural performance in our attentional task was also a sensitive variable for clustering patients, with more than 2 standard deviations in the case of RTs, and more than 3 for accuracy. Studies of attention in patients with MS have observed reduced speed of information processing, although accuracy is usually similar to that of healthy subjects, indicating that differences in accuracy between MS patients and controls are much smaller than differences in speed [4,37,39,78]. Our results confirm these results at least with RRMS patients. However, when accuracy is in part determined by time required for response, differences in accuracy between MS patients and controls could be as important as differences in speed for patients with many years of disease and a benign MS profile.

### Limitations and implications

Firstly, it should be noted that the cognitive abnormalities assessed in the present study using the Posner task might not be completely representative of the great variety and intensity of cognitive problems in MS. However, the main purpose of including the attentional Posner task is justified since attentional deficit and slowing of speed mental processing appears between the most impaired cognitive domains in this disease. Some variations of our experimental design may be taken into account in order to improve the sensitivity of our task, such as number, proportion and type of stimuli as inter-trial time, or response time. Further studies on new Posner version tasks may also help clarify this issue [79]. In addition, future studies employing a standard neuropsychological assessment could help to elucidate differences between specific cognitive domains. Conventional MRI measures are also required in order to obtain a more accurate interpretation of the relationship between MRI abnormalities and neuropsychological and neurophysiological cognitive function.

Another limitation in our study certainly comes from the small sample size examined in the two subgroups of MS, particularly in the benign group. Our patients may not be fully representative of the overall group of BMS subjects, and further studies including more patient groups apart from the MS spectrum (e.g. clinically isolated syndrome, secondary-progressive or primary forms of MS) are needed to verify the exact functional role of attentional and information processing in ERPs parameters. Despite this, our findings are in line with other studies that confirm the variability of the benign course of MS. Finally, in order to assess our results from a more global point of view, it would be particularly interesting to perform the cluster analysis including a larger set of clinical and paraclinical data in order to better establish the ecological validity of a cognitive profile-based classification of MS patients, as well as the relationship between ERP abnormalities and clinical evidence.

## Conclusions

The present study shows a number of significant differences between patients suffering from MS and age-matched control subjects, during completion of the visual-spatial Posner task with central cues. Our findings revealed varying levels of attentional and information processing in MS patients. When combined with behavioural methods, ERPs can help to elucidate the neuropsychological mechanisms underlying the cognitive impairments observed in this disease. Furthermore, after adjusting for subject age, disease duration in MS patients seems to be more closely related to alterations of short-latency of the ERP (N1 and P2) and early stage information processing. Abnormalities in the short-latency of the ERP may therefore be a good marker of specific information processing dysfunction in MS patients with low disability but a long disease duration.

Our findings indicate that both MS groups of our study have differentially altered patterns of cognitive processing, and that these patients may develop diverse degrees of altered cognitive processing. The characteristics of the groups including BMS patients provide empirical evidence about the heterogeneity of this disease course already suggested by electrophysiological [28,29], neuroimaging [80] and follow-up studies [7,81].

In terms of psychophysiological abnormalities, RRMS patients appear to form a more homogeneous clinical entity whereas BMS patients appear to form a more heterogeneous one. RRMS patients show mild/moderate alterations of cognitive processing, although some patients could even develop several deficits particularly related to slowing in information processing. The greater degree of abnormalities such as delays in information processing, orienting, and behavioural accuracy for BMS patients, may be explained by a longer disease duration and by a silent but continuous progression of disease.

In addition, the present study indicates the importance of differentiating MS groups according to cognitive capacities. Whether there is a benign form of MS continues to be a controversial issue [2,10,81]. Our findings also suggest, supported by evidence from neuroimaging and neuropathology studies, that some patients diagnosed with BMS could have a less benign course, therefore our results are in line with clinical evidence which suggests that the benign entity of MS can often be temporary and later become disabling.

## List of abbreviations

BMS: benign multiple sclerosis; CNV: contingent negative variation; CRs: correct responses; EDSS: expanded disability status scale; EEG: electroencephalography; ERPs: event-related potentials; MS: multiple sclerosis; RRMS: relapsing-remitting multiple sclerosis; RTs: reaction times.

## Competing interests

The authors declare that they have no competing interests.

## Authors' contributions

MVM, JJGR, PD, and GI contributed to the planning and preparation of the current study. MVM and JJGR conceived the experimental design and prepared the paradigm. The patients were selected by PD, MB and GI. JJGR and MVM were responsible for collecting the ERPs. JJGR analyzed the data, performed the statistical analysis, and drafted the manuscript. MVM, CMGG and GI contributed to the interpretation of results. MVM, CG and EV were intensely involved in the final revision of the manuscript. EV assisted with interpretation of the data, making sure that generalizations and claims about the data, so readily generated by the main author, were accurate. All authors read and approved the final manuscript.

## Pre-publication history

The pre-publication history for this paper can be accessed here:

http://www.biomedcentral.com/1471-2377/11/64/prepub
